# Development and validation of a physical literacy assessment questionnaire for college students

**DOI:** 10.3389/fpsyg.2025.1676038

**Published:** 2026-01-30

**Authors:** Lin Liu, Guang Hui Yang, Qi Zhang

**Affiliations:** School of Physical Education, Yanshan University, Qinhuangdao, China

**Keywords:** physical literacy, college students, questionnaire development, assessment tool, physical education

## Abstract

**Purpose:**

Physical literacy (PL) plays a vital role in promoting lifelong health, wellbeing, and sustained engagement in physical activity. This study aimed to develop and validate a physical literacy assessment questionnaire for college students (PLAQ-CS).

**Methods:**

This cross-sectional study employed a mixed-method sequential design to develop and validate the PLAQ-CS. Domains and preliminary items were generated through an extensive literature review and semi-structured interviews with 11 students. Fourteen experts evaluated item relevance and clarity to establish content validity. A total of 1,017 students participated in the quantitative phase to assess the reliability and validity of the questionnaire. Exploratory factor analysis (EFA) was used to refine the item pool, followed by confirmatory factor analysis (CFA) to examine factorial validity. Internal consistency was assessed using Cronbach's alpha.

**Results:**

The resulting PLAQ-CS comprises six domains: “physical skill,” “physical cognition,” “physical affectivity,” “physical habit,” “physical morality,” and “physical fitness.” The EFA showed 15 factors with an explanation of 70.844% of the total variance. CFA supported the six-factor structural model proposed by the researchers, evidenced by excellent model fit indices (GFI = 0.926, AGFI = 0.907, NFI = 0.828, RFI = 0.823, IFI = 0.819, TLI = 0.815, CFI = 0.844, RMSEA = 0.048). The Cronbach's Alpha coefficient for the PLAQ-CS was 0.956, with sub-questionnaires ranging from 0.780 to 0.896. The PLAQ-CS includes 47 items, all of which demonstrate good validity and reliability.

**Conclusion:**

The PLAQ-CS demonstrates scientific reliability and validity, making it suitable for assessing the PL levels of college students. Future research should focus on further validating this questionnaire across different countries and regions, with the goal of enhancing college students' PL and promoting the sustainable development of their physical and mental health.

## 1 Introduction

The importance of physical literacy (PL) in maintaining social stability (Barnett et al., 2023), fostering healthy lifestyles (Nguyen et al., 2020), and promoting individual physical and mental development (Caldwell et al., 2021) is increasingly recognized. Against this backdrop, a growing number of researchers suggest that PL levels could be used to describe the lifelong developmental process of individuals (Durden-Myers et al., 2022). As the backbone of national development, the physical and mental health of college students is of great concern to both the state and society. Due to misconceptions about physical exercise among college students, health issues such as overweight, obesity, and delayed motor development are becoming increasingly prevalent (Sheng et al., 2025). PL has emerged as a novel perspective for addressing these physical and mental health issues. Empirical evidence indicates that an individual's level of PL is positively associated with the development of healthy lifestyles and the enhancement of both physical and mental wellbeing (Carl et al., 2022). Moreover, higher levels of PL have been shown to improve physical and psychological health, enhance learning outcomes, and contribute to reducing healthcare costs (Blain et al., 2020; Mota et al., 2021; Riemann et al., 2021). Therefore, developing a scientifically sound and reasonable Physical Literacy Assessment Questionnaire for College Students (PLAQ-CS) holds significant practical importance and urgency for accurately assessing their PL.

The concept of PL directly influences the content of assessment. Whitehead originally defined PL as the combination of motivation, confidence, physical competence, knowledge, and understanding that enables individuals to value and engage responsibly in lifelong physical activity (Whitehead, 1993). Subsequent scholars have refined this concept, emphasizing lifelong engagement in physical activity through the integration of physical, cognitive, affective, and social capacities (Martins et al., 2021; Whitehead et al., 2018). The International Physical Literacy Association (IPLA) defines PL as the motivation, confidence, physical competence, knowledge, and understanding required for individuals to value and take responsibility for lifelong participation in physical activity (Young et al., 2019). It is evident that the concept of PL is gaining global recognition, with its value and significance widely disseminated. It should be noted that the diversity of the concept of PL results in variations in the relative weight of its constituent components. Overall, individuals lacking PL may not engage in physical activities, whereas those possessing PL will participate in physical activities persistently, even lifelong (forming a habit). Individuals continuously develop their confidence, abilities, knowledge, and understanding through participation in various sports activities, thereby valuing and taking ethics and responsibility.

Due to varying definitions of PL across countries and scholars, the development of standardized PL assessment tools and the scientific synthesis of research findings remain challenging (Edwards et al., 2017). Although various PL assessment tools have been developed internationally, most are standardized measures that lack adaptation for different age groups (Mandigo et al., 2019). For example, the Australian Physical Literacy Assessment Tool (PL-C Quest) evaluates four domains—physical, emotional, social, and cognitive—using 30 scenario-based self-report items (Diao et al., 2024). Similarly, the Canadian Assessment of Physical Literacy (CAPL) focuses on the physical, emotional, behavioral, and cognitive domains (Elsborg et al., 2021). In England, it is emphasized that measuring an individual's PL level should encompass affective, social, physical, and cognitive domains (Duncan et al., 2024). Brazil has explored the relationship between physical activity and PL, arguing that PL should not be limited to physical activity alone but should also include behavioral, physical health, social, and psychological dimensions (Filho et al., 2021). Portugal has designed a PL assessment questionnaire for adolescents aged 15–18, based on developmental stages (Cairney et al., 2019). Pakistan's assessment tools cover four aspects: motivation and confidence, daily behavior, physical competence, and knowledge and understanding (Hadier et al., 2024). In China, PL assessment indicators for children in grades 3–6 have been established, encompassing physical abilities, emotional factors, knowledge and understanding, and physical activity behaviors (YongKang and QianQian, 2022). Overall, there is inconsistency in the interpretation and content of PL assessment tools internationally, resulting in a lack of clarity and adaptability in evaluations. Research suggests that the physical and psychological development of children, adolescents, and adults exhibits distinct stage-specific characteristics (Blajwajs et al., 2023), which may necessitate differentiated emphases when assessing PL levels. Developing PL assessment tools specific to particular populations and cultural contexts may thus promote diversification of the PL construct and better reflect the diversity of physical and cultural practices across nations.

Given the current state of PL assessment tools and the practical needs for the physical and mental health development of contemporary college students, developing an assessment tool specifically for college students' PL is particularly important. At present, China is experiencing a significant deficit in PL assessment instruments specifically tailored for the college student demographic. These include the absence of a unified measurement framework, a need for more robust and validated methods, incomplete content dimensions, and limited practical applicability in real-world settings Choi et al., (2021); Ma et al., (2020). Existing assessment tools also have significant limitations, some of which stem from flaws in their development process, such as relying solely on literature reviews or modifying the assessment tools for PL in different countries Chen et al., (2020); Fang and Zhang, (2025); Luo et al., (2022). This can result in incomplete domains in the assessment tool or an inability to meet the needs of the individual being evaluated. Table 1 lists several assessment tools used to evaluate the PL of Chinese college students and adolescents. For example, China has developed the College Student Physical Literacy Questionnaire (CSPLQ), which encompasses only three domains: physical and behavioral, affective, and cognitive Luo et al., (2022). This may not accurately reflect college students' PL levels. As the concept of PL indicates, “the ability to value and engage responsibly in lifelong physical activity” constitutes a vital component Whitehead, (1993); Young et al., (2019). Therefore, habits, ethics, and physical fitness should be included as measurable elements in PL assessment tools. Research has also developed the Physical Literacy Index (PLI) for Chinese university students, assessing their PL levels across dimensions including motivation for sports, attitude toward sports, physical fitness, commitment to sports, physical activity level, appreciation of the body, confidence in sports, and willpower in sports (Wang et al., 2025). However, since these assessment tools rely solely on literature reviews to develop their content, they somewhat overlook individual experiences and perceptions of students, thereby failing to adequately address the challenges facing the professional learning development of Chinese university students. As a result, certain domains are omitted from PL assessment tools, preventing an accurate and scientific measurement of students' actual PL levels.

**Table 1:** Assessment tool for physical literacy of Chinese college students and adolescents.

**Tool**	**Assessment domains**	**Measurement method**	**Target audience ages**	**Study**
CSPLQ	Physical and behavioral. Affective. Cognitive.	Questionnaire	Chinese college students	Luo et al. (2022)
CAEPL	Physical activity. Knowledge of physical activity. Motor/sport skill. Behavior of physical activity. Physical fitness.	Questionnaire Online quiz On site skill display	Children aged 6–18 years old	Chen et al. (2020)
PLI	Motivation for sports. Attitude toward sports. Physical fitness. Commitment to sports. Physical activity level. Appreciation of the body. Confidence in sports. Willpower in sports.	Psychological scale measurements and field measurements	Chinese college students	Wang et al. (2025)
PLAQ	Physical competence. Affective domain. Knowledge and understanding. Behavior of physical activity.	Questionnaire	Chinese students in grades 3–6	YongKang and QianQian (2022)

To address these gaps, the present study aimed to develop and validate a comprehensive PLAQ-CS that captures the multidimensional nature of PL within Chinese higher education. Drawing upon both theoretical frameworks and empirical inquiry, this research sought to identify the core domains of PL and construct valid and reliable indicators encompassing physical, cognitive, affective, habitual, moral, and fitness dimensions. To achieve this aim, a three-stage research design was employed: (1) identification of PL domains through literature review and student feedback; (2) expert evaluation to establish content validity; and (3) psychometric testing to confirm structural validity. This study contributes to extending the conceptualization of PL to the university level and provides a culturally grounded framework for assessing and promoting students' holistic physical development in higher education settings.

## 2 Research methods

### 2.1 Participants

All the participants in this study were college students enrolled in various academic disciplines at universities and were divided into two groups. The first group comprised 11 participants (5 males and 6 females) aged between 18 and 22 years (mean age = 19.9 years, standard deviation = 1.13 years), enrolled in higher education institutions across various provinces and municipalities in China, their academic disciplines encompassing various fields such as literature, science, and engineering. The author conducted semi-structured interviews with 11 college students and collected interview data to establish a theoretical framework for college students' PL. The second group was comprised of 1,017 participants who completed the initial PLAQ-CS. Among the 1,017 participants, their academic disciplines encompassed literature, science, engineering, education (including physical education), economics, management, and other fields. Of the 1,017 questionnaires, invalid responses were excluded (reasons for exclusion included convergent answers or logical inconsistencies in response options). Ultimately, 940 valid questionnaires were returned (validity rate: 92.4%). The final sample (*n* = 940) was randomly divided into two subsamples using the SPSS random case-selection function. Subsample 1 (*n* = 462) comprised the calibration sample (247 males and 215 females). Participants were undergraduate, master's, and doctoral students from a comprehensive university in China (undergraduates = 369, master's students = 65, doctoral students = 28; mean age = 23.28 years, standard deviation = 2.15 years). Item analysis and exploratory factor analysis (EFA) were conducted for the initial PLAQ-CS. Subsample 2 (*n* = 478) served as the validation sample (258 males, 220 females, undergraduates = 390, master's students = 72, doctoral students = 16; mean age = 22.84 years, standard deviation = 1.96 years). This sample was used to conduct confirmatory factor analysis (CFA) and supplement psychometric assessments, including internal consistency reliability and construct validity. The study was conducted in accordance with the guidelines of the Declaration of Helsinki and approved by the Institutional Review Board of the School of Physical Education, Yanshan University. All the participants provided written informed consent.

### 2.2 Procedure

**Figure 1:**
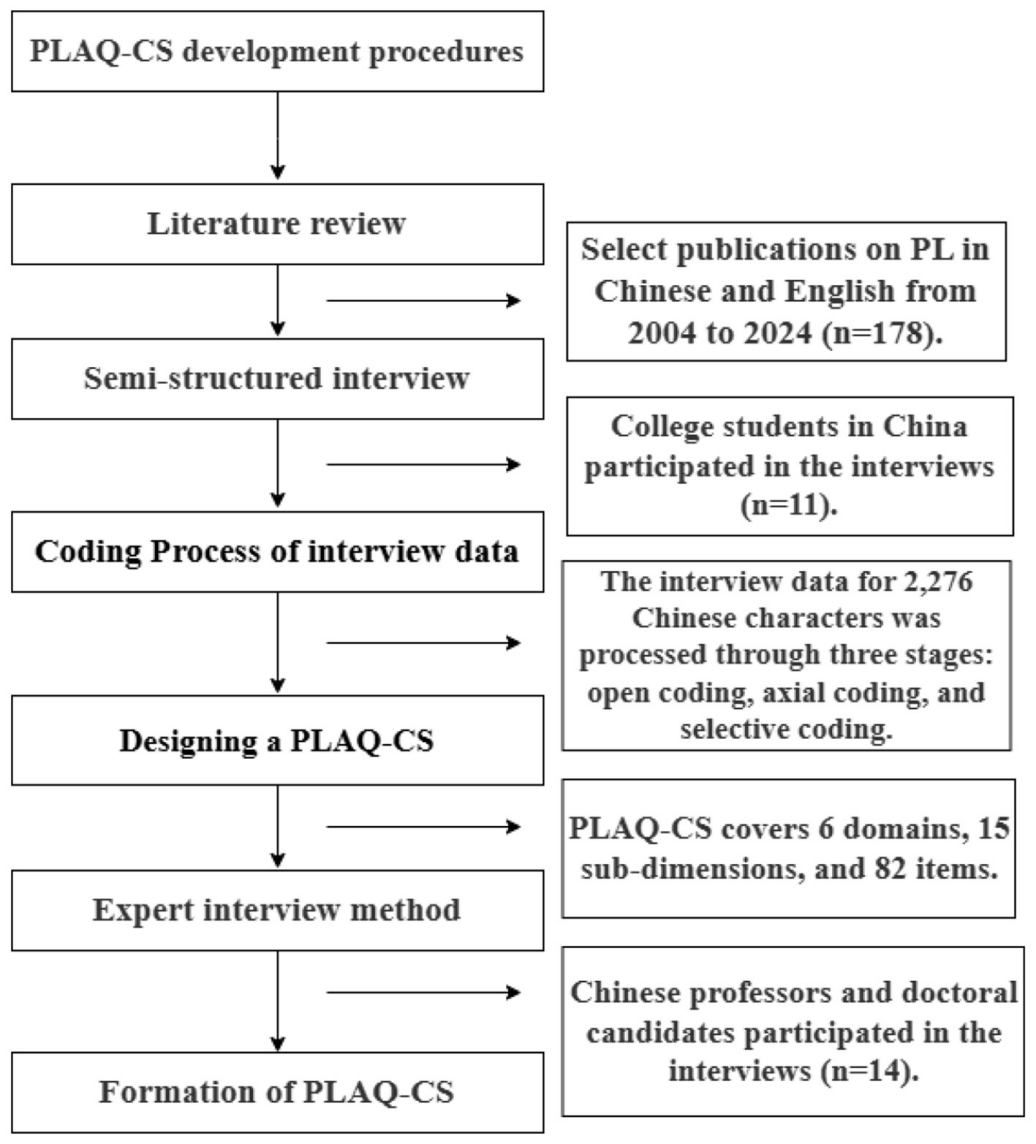
Flowchart for developing the physical literacy assessment questionnaire for college students.

This study employed a combined qualitative and quantitative method to develop and validate the PLAQ-CS. Specifically, building upon theoretical research (literature review), we extensively solicited opinions and suggestions from stakeholders and relevant parties (semi-structured interviews). Subsequently, we integrated insights and findings from both methodologies to construct the domains and dimensions of the PLAQ-CS. This mixed-methods research design emphasizes deductive reasoning at the theoretical level and inductive reasoning based on opinions from various sources (Fan et al., 2018; Ralph and MacPhail, 2015; Wang et al., 2018). This enhances the representativeness and comprehensiveness of the research content. The specific research procedure is shown in Figure 1.

#### 2.2.1 Literature review

The literature review aimed to clarify the fundamental content structure of PL, clearly identifying its concepts, characteristics, appropriate populations, and various assessment tools. Key search terms included “physical literacy,” “college students,” and “physical literacy assessment tool.” Only publications in English and Chinese from 2004 to 2024 were included to ensure contemporary relevance (a total of 178 articles). To minimize bias, all pertinent publications were considered regardless of research methodology (qualitative, survey, systematic review, or quantitative), article type (theoretical or empirical), or source. The literature was thoroughly analyzed, and discussions with field experts were conducted until consensus was reached regarding the definition of PL.

#### 2.2.2 Semi-structured interview

Once the concept of PL was established, researchers began conducting semi-structured interviews with college students. This study did not predetermine the number of interviewees at the outset; instead, it operated on the principle of data saturation. That is, when the same content is repeatedly observed or heard, it becomes evident that further data collection will yield no additional insights, indicating that “data saturation” has been reached. At this point, the interviews are concluded. After analyzing data from the tenth college student interviewee, the researcher found no new elements of PL emerging. Adhering to the principle of rigor, the researcher conducted one additional interview with another college student. Analysis of this data also yielded no new elements. Therefore, the final number of college student interviewees was determined to be 11. Researchers conducted interviews with 11 college students using both WeChat and face-to-face methods. The participants were enrolled in universities across various provinces and municipalities in China and completed an open-ended questionnaire containing two questions: (a) What do you think PL should encompass? and (b) What methods do you think can be used to cultivate students' PL? After the interviews, the authors applied Grounded Theory (GT), as proposed by Glaser and Strauss, to code the interview data. Coding involves the systematic organization of interview data and typically includes open coding, axial coding, and selective coding (Corbin and Strauss, 2014).

#### 2.2.3 Coding process of interview data

In the open coding phase, the researcher extracts concepts from a large volume of raw interview data, ensuring that these concepts are as close as possible to the original information. This process focused on identifying specific words and phrases that directly reflect the components of “physical literacy” for college students.

Following this, axial coding was applied. Researchers inductively organized the concepts formed during open coding to identify subcategories (second-level dimensions), then examined the relationships among these subcategories to establish main categories (first-level dimensions). Throughout this process, researchers repeatedly compared the main categories with the initial concepts generated during the open coding phase, extracting core concepts related to PL from the raw data while eliminating contradictory labeled nodes.

In the selective coding stage, the researcher established relationships between the main categories and subcategories identified in axial coding to build, present, and explain specific behaviors of the research subjects. The domains identified through coding were then compared with the literature review results to validate the findings. To gain a deeper understanding of domain structure and item development, the authors also reviewed existing PL assessment questionnaires, such as the PL-C Quest (Diao et al., 2024). Based on the literature and coding analysis, preliminary domains for college student PL were developed, and an item bank was created.

#### 2.2.4 Designing the PLAQ-CS

Based on literature reviews and student interview data, the initial PLAQ-CS draft comprises 6 domains, 15 sub-dimensions, and 82 items. The initial 82 items of the PLAQ-CS were grouped into six sub-questionnaires: “physical skill (PS)” (12 items), “physical cognition (PC)” (17 items), “physical affectivity (PA)” (11 items), “physical habit (PH)” (11 items), “physical morality (PM)” (15 items), and “physical fitness (PF)” (16 items) (Appendix 1). All items were rated on a five-point Likert scale ranging from “Strongly Disagree (representing the number 1)” to “Strongly Agree (representing the number 5)”, with a neutral midpoint, enabling nuanced measurement of latent constructs (Groves et al., 2011). Content validity of the questionnaire items was assessed through expert interviews.

#### 2.2.5 Expert interview method

In this study, we employed the expert interview method, a technique used to improve, investigate, and develop items (Willis, 2004). This method involves consulting specialists in specific fields to gather information, aiming to obtain the most reliable consensus opinions from the interviewed group. Fourteen professors and doctoral candidates specializing in school physical education in China (Table 2) were selected to participate in the initial content validity testing of the questionnaire. These experts were chosen based on their previous publications on the topics of “physical literacy” and “physical education.” We emailed an online survey link from wjx.cn to the fourteen experts. The questionnaire consisted of the initial PLAQ-CS, which includes 6 domains, 15 sub-dimensions, and 82 randomly ordered survey items (Appendix 2). Experts were first asked to evaluate whether the 6 domains included in the questionnaire were appropriate and to provide relevant recommendations. Subsequently, they assessed whether the 15 sub-dimensions under the 6 domains remained relevant to their respective domains and marked their agreement or disagreement for each item within each sub-dimension. Experts need to provide revision suggestions for items they disagree with. At this stage, the degree of consensus among the experts regarding the alignment of each item with its domain was calculated. Items agreed upon by at least 80% of experts were considered to have acceptable content reliability and validity (Groves et al., 2011). Based on expert feedback, the wording of some items was revised because they were either unclear or not relevant to the intended domains.

**Table 2:** Expert personal information form.

**Expert**	**Title**	**Research focus**	**Identity**
A01	Professor	School Physical Education	University faculty
A02	Professor	School Physical Education	University faculty
A03	Professor	Physical Education Theories	University faculty
A04	Professor	School Physical Education	University faculty
A05	Professor	Physical Education	University faculty
A06	Associate Professor	Sociology of Sport	University faculty
A07	Associate Professor	School Physical Education	University faculty
A08	Associate Professor	Physical Education Theories	University faculty
A09	Associate Professor	Physical Education Theories	University faculty
A10	Associate Researcher	School Physical Education	University faculty
A11	Lecturer	School Physical Education	University faculty
A12	Lecturer	School Physical Education	University faculty
A13	Doctor	School Physical Education	Doctoral candidate
A14	Doctor	School Physical Education	Doctoral candidate

### 2.3 Data analysis

After developing the initial PLAQ-CS, EFA and CFA were conducted using SPSS 26.0 and AMOS 24.0 software to establish the final assessment questionnaire. Subsequently, reliability analysis (i.e., internal consistency among items) was performed using SPSS 26.0 to ensure the measurement questionnaire's reliability.

#### 2.3.1 Exploratory factor analysis (EFA)

The researcher used SPSS 26.0 to conduct the Bartlett's test of sphericity and the KMO numerical test on the initial questionnaire, which will verify the applicability of the variables in this paper to factor analysis. It is generally accepted that factor analysis is well suited when (KMO greater than 0.9, sig ≤ 0.01). An EFA was conducted to identify potential common factors. Using principal component extraction, the number of common factors was determined based on the eigenvalue (>1) and the scree plot. Since the correlation between the two factors was not close to zero, promax rotation was used as suggested by Meyers and colleagues (Meyers et al., 2016). Get the factor loadings of the rotated items. Items were deleted if the rotated factor loadings were below 0.40 or if there were cross-loadings (i.e., the items loaded close together on both factors).

#### 2.3.2 Confirmatory factor analysis (CFA)

Using the items retained from the EFA, researcher conducted a CFA using AMOS.24.0 to test for factorial validity, thus demonstrating the structural validity of the questionnaire obtained from the EFA results. According to Meyers et al. fit indices include Root Mean Square Error of Approximation (RMSEA), Comparative Fit Index (CFI), Tucker-Lewis Index (TLI), and Cardinality/Degree of Freedom (χ^2^/*df*) to test the overall fit of the model to the data (Meyers et al., 2016). Numerous scholars have argued that the degree of model fit requires observing the RMSEA value, which should generally be less than 0.08, but less than 0.1 is also acceptable (Lei and Lomax, 2005). To further corroborate the reliability of the factorial validity analysis, we also examined the values of Adjust Goodness-Of-Fit Index (AGFI), Normed Fit Index (NFI), Relative Fit Index (RFI), Incremental Fit Index (IFI), and Goodness of Fit Index (GFI) simultaneously. It is generally recognized that AGFI, NFI, RFI, CFI, TLI, IFI, and GFI values should be greater than 0.9, but individual indicators higher than 0.8 and slightly lower than 0.9 are acceptable (Browne and Cudeck, 1992; Sharma et al., 2005).

#### 2.3.3 Reliability analysis

After excluding items with low factor loadings or high residual covariances, Cronbach's alpha was calculated using SPSS 26.0 to assess the internal consistency of the items within each outcome domain and the questionnaire as a whole. Cronbach's alpha is commonly used to indicate the reliability of a measurement (Cohen et al., 2013). Values of Cronbach's alpha between 0.7 and 0.8 indicate acceptable reliability, while values between 0.8 and 0.9 suggest an ideal questionnaire.

## 3 Research results

### 3.1 Coding process results

#### 3.1.1 Open coding results

Following the completion of open coding, all interview data from participants were coded to generate initial concepts related to PL. Ultimately, 15 non-duplicate initial concepts were identified (Table 3). Due to the extensive nature of the interview data, Table 3 presents only a portion of the open coding process.

**Table 3:** Open coding content.

**Interview materials**	**Label**	**Initial concept**
1) Possess excellent athletic ability, such as mastering fundamental movements like running, jumping, and throwing.	Physical activity ability.	Fundamental sport skills.
2) Master one or two sports you genuinely enjoy, such as basketball, badminton, or swimming.	Sports skills.	Specialized sport skills.
3) Understand the rules and key techniques of the sport you practice, and adhere to these rules during competitions and games.	Understand knowledge. Follow the rules.	Understand sports knowledge. Apply sports knowledge. Ethics.
4) How to avoid injuries during exercise, and what to do if you sprain your ankle.	Understand knowledge. Apply knowledge.	Understand sports knowledge. Apply sports knowledge.
5) Stay updated on sports news and information, and be aware of sports-related events happening around you.	Get Sports News. Get sports information.	Acquiring sports knowledge.
6) Honestly, most sports are pretty similar. I believe that with dedicated learning and consistent practice, anyone can master them. I played basketball in middle school, and even now when I play with students from the sports college, it's still quite competitive.	Believe in yourself. Sports skills. Stay active in sports. Tenacious struggle.	Confidence in sport. Specialized sport skills. Sustained exercise habits. Automatic exercise habits. Spirit.
7) If you want to lose weight, build muscle, or tone up, just randomly running or lifting weights is inefficient—you need to understand the basic principles.	Purpose of sport. Sports knowledge.	Motivation for sport. Understand sports knowledge. Apply sports knowledge.
8) Don't get discouraged after losing a game, don't blame teammates, don't make excuses. Enjoy the process and learn from the experience.	Maintain composure. Stay united. Respect teammates.	Spirit. Ethics. Character.
9) I believe one should be physically strong, not prone to illness, and neither too thin nor too overweight, as either extreme gives an unfavorable impression.	Strong. Resistance. Physique.	Physical quality. Physical functioning. Physical form.

#### 3.1.2 Axial coding results

Subsequently, axial coding was performed with the primary objective of screening initial concepts to establish subcategories (second-level dimensions), thereby determining the main categories (first-level dimensions) that emerge. The primary category is derived by integrating the connotations of the initial concepts with the subcategories. Thus, the main categories, subcategories, and initial concepts are interrelated. These categories primarily exhibit the following characteristics: core attributes that can be correlated with other data; the ability to explain most of the behavior within the data content; and relevance and meaningfulness in relation to other variables. Based on these principles and through continuous comparison of concepts, the principal axial coding results reveal the multidimensional structure of PL. This study proposes that the components of college students' PL consist of 6 main domains: physical skill (PS), physical cognition (PC), physical affectivity (PA), physical habit (PH), physical morality (PM), and physical fitness (PF). Within these 6 domains, there are 15 secondary dimensions. The PS domain includes fundamental sport skills (FSK) and specialized sport skills (SSK). The PC domain covers physical knowledge acquisition (PKAC), physical knowledge comprehension (PKC), and physical knowledge application (PKAP). The PA domain includes physical motivation (PMO) and physical confidence (PCO). The PH domain comprises habit stabilization (HS) and habit automation (HA). The PM domain encompasses physical spirit (PSP), physical ethics (PE), and physical character (PCH). The PF domain includes physical quality (PQ), physical functioning (PFU), and physical form (PFO) (Table 4).

**Table 4:** Axial coding content.

**First-level dimensions**	**Second-level dimensions**	**Screening and classification of initial concepts**
PS	FSK	Fundamental sport skills
	SSK	Specialized sport skills
PC	PKAC	Acquiring sports knowledge
	PKC	Understand sports knowledge
	PKAP	Apply sports knowledge
PA	PMO	Motivation for sport
	PCO	Confidence in sport
PH	HS	Sustained exercise habits
	HA	Automatic exercise habits
PM	PSP	Spirit
	PE	Ethics
PCH	Character
PF	PQ	Physical quality
	PFU	Physical functioning
PFO	Physical form

PS, physical skill; PC, physical cognition; PA, physical affectivity; PH, physical habit; PM, physical morality; PF, physical fitness; FSK, fundamental sport skills; SSK, specialized sport skills; PKAC, physical knowledge acquisition; PKC, physical knowledge comprehension; PKAP, physical knowledge application; PMO, physical motivation; PCO, physical confidence; HS, habit stabilization; HA, habit automation; PSP, physical spirit; PE, physical ethics; PCH, physical character; PQ, physical quality; PFU, physical functioning; PFO, physical form.

#### 3.1.3 Selective coding results

Selective coding primarily focuses on explaining the relationships between main categories and subcategories, specifically examining the behavioral interactions among physical skill (PS), physical cognition (PC), physical affectivity (PA), physical habit (PH), physical morality (PM), physical fitness (PF), and PL (Table 5). After defining these domains and dimensions, the researchers developed 82 project items based on these parameters. It is important to note that these domains and dimensions not only encompass the content found in PL assessment tools proposed by scholars and organizations but also expand upon them further.

**Table 5:** Selective coding content.

**Domains**	**The implications of relational structures**
PS	PS serve as both the fundamental means for college students to participate in physical activities and a crucial component of their PL development. It can be said that only by mastering these skills can individuals experience the diverse insights gained through physical activities, thereby enabling their PL to truly form and develop.
PC	The core essence of PC lies in the integrated acquisition, comprehension, and application of physical education knowledge by individuals. More specifically, it encompasses an individual's understanding of how, when, and why to engage in physical activities, along with their recognition of the benefits of sports participation.
PA	PA serve as the driving force behind the cultivation and development of PL, determining an individual's tendency to approach or avoid the environment, as well as the level of effort they exert during the development of PL.
PH	PH address the challenge of translating the cultivation and development of PL into practice. They are the foundation for maintaining and advancing PL, and the key to its sustainable development.
PM	PM enables individuals to strive to overcome various internal and external factors during participation in physical activities. It also empowers college students to rationally navigate multiple relationships with others and external entities, thereby serving as a spiritual foundation for the development of one's PL.
PF	PF serves as the foundation within the framework of PL, providing the essential basis for college students to engage in physical activities.

PS, physical skill; PC, physical cognition; PA, physical affectivity; PH, physical habit; PM, physical morality; PF, physical fitness.

### 3.2 Expert interview results

According to expert feedback, ten of the 82 items in the questionnaire do not meet the requirements for content validity and reliability. Because experts have low agreement (i.e., <80%) on these items. The problems with these items are respectively that the presentation is not clear or that the presentation contains crossover with the various domains. Detailed expert comments (Appendix 3). For example, the item “My physical activity capacity is strong.” made it difficult to define the range of “strong”, so the formulation of the item was replaced. Whereas “I know the practice methods for a specific sports technique.” is more controversial when placed under the domain of physical skill (PS), it may be more of an item under the domain of physical cognition (PC), which intersects with the domains of PS and PC. Minor wording changes were made to the remaining items based on expert feedback on the clarity of the items. Ultimately, the 82 items in the preliminary initial PLAQ-CS were updated and revised.

### 3.3 Bartlett's test of sphericity and KMO index

Table 6 presents the results of the Bartlett's test of sphericity and the KMO value test for the core domains of PL, which concluded that the variables in this study were well suited for factor analysis (KMO = 0.923, sig = 0.00).

**Table 6:** KMO measures and Bartlett's test of sphericity.

**KMO quantity of sample suitability**		**0.923**
	Approximate chi-square value	32656.384
Bartlett's test of sphericity	degrees of freedom	3,321
	significance	0.000

### 3.4 Exploratory factor analysis (EFA) results

The EFA results are presented in Table 7, where the common factors with the highest significance were selected based on their eigenvalues. Fifteen co-factors were identified as the primary factors, accounting for a cumulative contribution of 70.844%. The promax rotation method was applied to the factor loading matrix to clarify the common meaning of the factors. The first through fifteenth factors were habit stabilization (HS), physical ethics (PE), physical character (PCH), specialized sport skills (SSK), physical form (PFO), physical knowledge application (PKAP), physical confidence (PCO), physical knowledge comprehension (PKC), fundamental sport skills (FSK), physical quality (PQ), physical spirit (PSP), habit automation (HA), physical motivation (PMO), physical functioning (PFU), and physical knowledge acquisition (PKAC). During this process, items with loadings less than 0.4 were eliminated, resulting in the PLAQ-CS (Table 8) comprising 47 screened items.

**Table 7:** Total variance explained.

**Factors**	**Initial eigenroots**			**Rotated factor loadings**		
	**Totals**	**Percentage of variance explained**	**Cumulative percentages**	**Totals**	**Percentage of variance explained**	**Cumulative percentages**
HS	27.530	33.574	33.574	10.312	12.576	12.576
PE	6.505	7.932	41.506	8.742	10.662	23.238
PCH	4.164	5.078	46.584	5.660	6.902	30.140
SSK	2.662	3.247	49.831	4.791	5.843	35.983
PFO	2.492	3.039	52.870	4.637	5.655	41.638
PKAP	2.147	2.619	55.489	3.458	4.217	45.855
PCO	1.923	2.345	57.834	3.445	4.202	50.057
PKC	1.848	2.254	60.088	3.237	3.948	54.005
FSK	1.506	1.836	61.924	2.599	3.169	57.174
PQ	1.381	1.684	63.608	2.352	2.868	60.042
FSP	1.339	1.633	65.241	2.174	2.652	62.694
HA	1.254	1.529	66.770	1.895	2.311	65.004
PMO	1.159	1.413	68.183	1.888	2.303	67.307
PFU	1.104	1.347	69.530	1.493	1.821	69.128
PKAC	1.077	1.314	70.844	1.407	1.716	70.844

FSK, fundamental sport skills; SSK, specialized sport skills; PKAC, physical knowledge acquisition; PKC, physical knowledge comprehension; PKAP, physical knowledge application; PMO, physical motivation; PCO, physical confidence; HS, habit stabilization; HA, habit automation; PSP, physical spirit; PE, physical ethics; PCH, physical character; PQ, physical quality; PFU, physical functioning; PFO, physical form.

**Table 8:** Physical literacy assessment questionnaire for college students.

**Domains**	**Second dimensions**	**Items**	**Degree of agreement**
(A) PS	A1 FSK	(A11) I can easily participate in gym class by practicing sports techniques or playing games.	1 2 3 4 5
		(A12) I can perform basic walking, running, jumping, throwing and bracing movements.	1 2 3 4 5
		(A13) When exercising, I can control the speed and power of my body movements, etc.	1 2 3 4 5
	A2 SSK	(A21) I have mastered an athletic skill (not limited to multi-sport items such as soccer, basketball, etc.).	1 2 3 4 5
		(A22) In physical education classes, teachers or classmates sometimes praised my athletic skill level.	1 2 3 4 5
		(A23) I can make demonstrations of certain sports techniques.	1 2 3 4 5
		(A24) I can tell my peers the essentials of certain sports techniques.	1 2 3 4 5
		(A25) I can score good or above on sports techniques I have learned in physical education exams.	1 2 3 4 5
(B) PC	B1 PKAC	(B11) I will proactively consult books, reference materials, and other texts to learn about sports and physical education.	1 2 3 4 5
		(B12) I can watch sports competitions to learn about techniques and tactics of sports items.	1 2 3 4 5
		(B13) I can take the initiative to I can learn the rules of sports.	1 2 3 4 5
	B2 PKC	(B21) I can understand the referee's decisions in sports competitions.	1 2 3 4 5
		(B22) I know that I should avoid exercising in environments that are not conducive to good health (e.g., hazy days).	1 2 3 4 5
		(B23) I can reflect on the technical and tactical mistakes made by myself or my team in competitions.	1 2 3 4 5
	B3 PKAP	(B31) I can apply what I have learned in my daily workouts.	1 2 3 4 5
		(B32) I can apply my knowledge of sports in competitions techniques and tactics.	1 2 3 4 5
		(B33) I am able to apply 1-2 skills scientifically and rationally to improve cardiorespiratory endurance, muscular strength and flexibility (aerobic running, deep squats, etc.).	1 2 3 4 5
(C) PA	C1 PMO	(C11) I want to master a motor skill through my physical education class.	1 2 3 4 5
		(C12) A big reason I work hard in class is to get good grades.	1 2 3 4 5
		(C13) I work hard to improve my level of motor skills to be praised by my teachers and peers.	1 2 3 4 5
	C2 PCO	(C21) I don't worry about quizzes in my physical education class.	1 2 3 4 5
		(C22) In physical education competitions, I think I can perform well.	1 2 3 4 5
		(C23) I am not afraid to participate in a variety of activities related to physical education and sports.	1 2 3 4 5
(D) PH	D1 HS	(D11) If nothing else, sports is something I have to do.	1 2 3 4 5
		(D12) I play sport even when there is no physical education class (or exam).	1 2 3 4 5
		(D13) I find a way to play sport even when it is difficult.	1 2 3 4 5
	D2 HA	(D21) Sports are a part of my daily life.	1 2 3 4 5
		(D22) I don't need anyone's (teachers, parents) urging to participate in sport.	1 2 3 4 5
(E) PM	E1 PSP	(E11) I am not afraid of the sport challenges in classroom learning.	1 2 3 4 5
		(E12) I always persevere when I encounter difficulties or bottlenecks in learning sports skills.	1 2 3 4 5
		(E13) I always do my best when participating in sports.	1 2 3 4 5
	E2 PE	(E21) I follow the rules when participating in all kinds of sports.	1 2 3 4 5
		(E22) I can be honest in taking tests and do not cheat during sports tests.	1 2 3 4 5
		(E23) I think it is not right to cheat in sports competitions.	1 2 3 4 5
		(E24) When playing sports, I can show respect for my peers and opponents.	1 2 3 4 5
	E3 PCH	(E31) I will take the initiative to help my classmates with poorer athletic performance to learn.	1 2 3 4 5
		(E32) Whether I win or lose, I look at the results of the game correctly.	1 2 3 4 5
(F)PF	F1 PQ	(F11) I have never felt powerless when playing sports.	1 2 3 4 5
		(F12) I can react quickly when facing unexpected problems during sports.	1 2 3 4 5
		(F13) I find that my body has become more coordinated after I learn in the sports classroom.	1 2 3 4 5
	F2 PFU	(F21) I have never been unresponsive after I have played sports.	1 2 3 4 5
		(F22) I have never experienced fainting while playing sports.	1 2 3 4 5
		(F23) After participating in sports, I find that my immunity has improved (e.g., I get sick less often).	1 2 3 4 5
	F3 PFO	(F31) My body mass index (BMI) is within the standard range (BMI = weight (kg)/height (m)^2^ [18.5 ≤ standard ≤ 23.9]).	1 2 3 4 5
		(F32) I am satisfied with my physical form.	1 2 3 4 5
		(F33) I believe my physical form meets the requirements for participating in sports.	1 2 3 4 5
		(F34) During sport, I consider my physical form capable of supporting me in completing technical movements.	1 2 3 4 5

The PLAQ-CS consists of 47 items scored from 1 to 5 (There are five options in the Degree of Agreement: 1 = Strongly disagree, 2 = Disagree, 3 = Neutral, 4 = Agree, 5 = Strongly agree). The total score ranges from 47 to 235, and higher scores indicate a high level of physical literacy. A = Physical skill, B = Physical cognition, C = Physical affectivity, D = Physical habit, E = Physical morality, F = Physical fitness.

### 3.5 Confirmatory factor analysis (CFA) results

**Figure 2:**
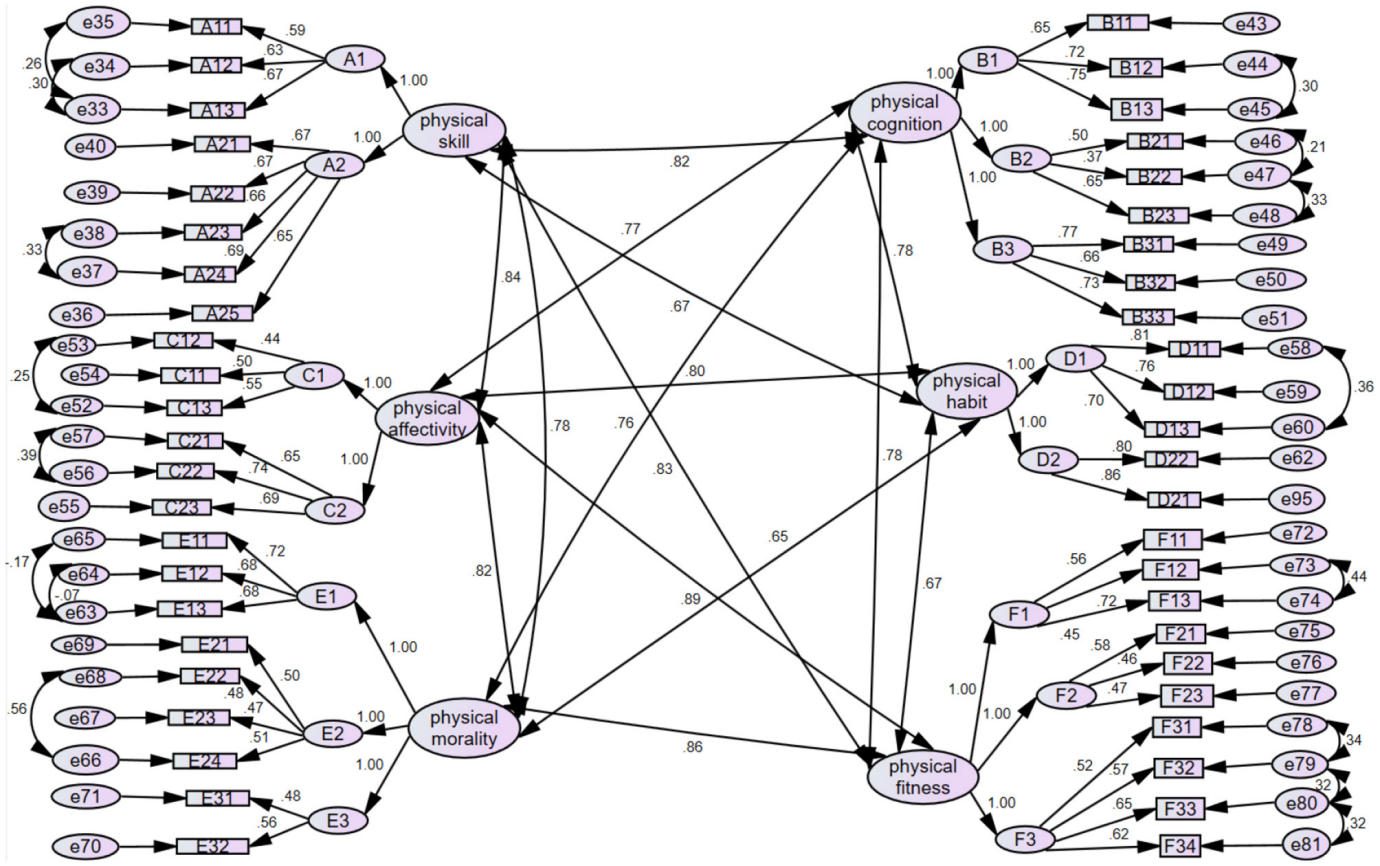
Structural equation model of the physical literacy assessment questionnaire for college students.

After completing EFA, a CFA was conducted on the PLAQ-CS. The CFA showed (Table 9) that the six indicators of the initial model of PLAQ-CS, AGFI, NFI, RFI, IFI, TLI and CFI, did not meet the fitness criteria. This indicates that the model fit (goodness of fit) of the structural equation of APL-CS is bad, and therefore the model needs to be corrected. Adopting the Bootstrapping method recommended by Bollen and other scholars (Bollen and Stine, 1992), the model goodness-of-fit test (χ^2^) value is corrected, and the results of the model fitness before and after correction are shown in Table 9, which shows that the AGFI indicator fitness index is found to be in line with the fitness model standard after correction. The five indicators of NFI, RFI, IFI, TLI, and CFI are all higher than 0.8, although they do not meet the standard. Therefore, all the fitness indices of the second-order six-factor structural model of college students' PL are in line with the standard values of the fitness model, and the structural model fits well, with good structural validity (Figure 2).

**Table 9:** Confirmatory factor analysis results of the physical literacy assessment questionnaire for college students.

**Statistical test**	**Criteria or thresholds for adaptation**	**Before model correction**	**After model correction**
RMSEA	<0.08 (<0.05 excellent, <0.08 virtuous)	0.0891	0.048
GFI	>0.90	0.907	0.926
AGFI	>0.90	0.879	0.907
NFI	>0.90	0.701	0.828
RFI	>0.90	0.579	0.823
IFI	>0.90	0.752	0.819
TLI	>0.90	0.623	0.815
CFI	>0.90	0.750	0.844
χ2 / df	Between 0 and 3 (<5 is acceptable)	2.839	2.376

### 3.6 Reliability testing results

Following the completion of the CFA, we conducted internal consistency testing (reliability testing) for the questionnaire and its items. The results indicate that each domain and the overall questionnaire demonstrate relatively high internal consistency. Specifically, the questionnaire's Cronbach's Alpha values for the six domains—physical skill (PS), physical cognition (PC), physical affectivity (PA), physical habit (PH), physical morality (PM), and physical fitness (PF)—are 0.863, 0.868, 0.780, 0.896, 0.834, and 0.827, respectively. The overall questionnaire's Cronbach's Alpha value was 0.956 (Table 10).

**Table 10:** Reliability statistics.

**Domains**	**Cronbach's alpha**	**N of items**
Questionnaire	0.956	47
PS	0.863	8
PC	0.868	9
PA	0.780	6
PH	0.896	5
PM	0.834	9
PF	0.827	10

PS, physical skill; PC, physical cognition; PA, physical affectivity; PH, physical habit; PM, physical morality; PF, physical fitness.

### 3.7 Validity testing results

This study employed expert interviews, inviting 14 experts to refine, modify, and improve all assessment indicators and items within the PLAQ-CS. Consequently, the PLAQ-CS demonstrates strong content validity. Furthermore, CFA results indicate that the PLAQ-CS exhibits robust structural validity. In summary, the PLAQ-CS possesses excellent validity.

## 4 Discussion

This study aimed to develop the PLAQ-CS. Unlike previous research, it innovatively combined a literature review with semi-structured interviews of college students to create the PLAQ-CS. We believe this questionnaire will make a valuable contribution to ongoing research on PL.

### 4.1 Development of the PLAQ-CS in China

Currently, Chinese scholars have developed a PL assessment tool for college students. This evaluation questionnaire (CSPLQ) encompasses three domains: physical and behavioral, affective, and cognitive. It also includes seven dimensions (motor skills, physical activity, perceptions of healthy living, perceptions of physical activity, motivation to engage in physical activity, and confidence to engage in physical activity, comprising a total of 38 items (Luo et al., 2022). However, the CSPLQ has limitations in two aspects. Firstly, PL is multidimensional (Pastor-Cisneros et al., 2025), so relying solely on the physical, affective, and cognitive domains cannot fully reflect or measure all aspects of college students' PL. Secondly, the design concept of CSPLQ has certain shortcomings because it relies exclusively on literature review to determine the domains and dimensions of the evaluation tool. This approach may overlook some PL component indicators that fall outside the theoretical framework, resulting in an assessment tool that cannot comprehensively, precisely, and accurately measure the PL of college students. To address these issues, the PLAQ-CS employs a comprehensive research methodology that combines literature review with interview data to identify the constituent elements of college students' PL and construct a robust framework. It further enriches and broadens the field of PL research.

The PLAQ-CS encompasses a broader range of domains than existing PL assessment tools, incorporating domains such as morality, habits, and physical fitness. Including these three domains in PL assessments more effectively highlights the true essence and value of PL. Physical morality represents the ethical standards and principles that students should uphold when participating in physical activities. This means individuals must demonstrate perseverance, adhere to rules and regulations, and embody integrity and kindness. It reflects a deeply humanistic sentiment and serves as a crucial foundation for the development of PL. Physical habits extend beyond mere exercise routines, emphasizing a lifestyle, attitude, and spiritual pursuit that embody a continuous, dynamic developmental process. This is essential for nurturing PL and promoting lifelong participation in sports. Physical fitness is a key indicator of an individual's level of PL. Neglecting this element not only diminishes the value of other components—such as skills, cognition, affection, habits, and morality—but also weakens the intrinsic connections among them. Moreover, the level of physical fitness directly influences the development of an individual's PL. In summary, the PLAQ-CS not only enriches the theoretical framework of PL with Chinese cultural characteristics but also offers valuable Chinese insights and solutions to global PL research.

### 4.2 Factorial reliability and validity of the questionnaire

This study aimed to validate the PLAQ-CS using two analytic methods (EFA and CFA) in one study. Sample size is important for EFA and CFA (Meyers et al., 2016). In the initial EFA validation analysis of the questionnaire, the number of participants exceeded five times the total number of items. However, when the final questionnaire was screened, the CFA had an approximate participant-to-item ratio of 10:1, which greatly enhanced the accuracy of the EFA vs. the CFA (DeVellis and Thorpe, 2021). Therefore, we believe that the sample size is sufficient to yield statistically significant results. The CFA and EFA results were consistent with the structural equation modeling of the PLAQ-CS, providing strong evidence for the questionnaire's structural validity. Cronbach's alpha coefficients for the entire questionnaire and the six domains exceeded 0.70, with overall reliability above 0.90, and five domains above 0.80, indicating acceptable reliability both within and across the questionnaire (Meyers et al., 2016).

It is worth noting that some negatively worded items in the initial questionnaire were removed due to their low factor loadings, such as “I think my physical form is a burden during exercise.” Generally, negatively worded items are included in questionnaires to reduce the response bias (Groves et al., 2011). However, research has shown that this approach can lead to ambiguous results and low reliability (Roszkowski and Soven, 2010). Future research should further explore the necessity of including negatively worded items in questionnaires. In conclusion, the PLAQ-CS developed by the authors is a reliable and valid tool that can be used in future teaching practice and academic research. However, it is important to revalidate the questionnaire before applying it to other countries or subject areas, as the physical and mental characteristics of college students as well as their educational systems may differ across regions. For example, American schools tend to have more classroom-based teaching, including intergroup discussions, expert lectures, questions, skill demonstrations, and practical exercises (Raleigh et al., 2018), there are significant differences between this classroom teaching format and that of Chinese schools.

### 4.3 Implications of using the questionnaire

The PLAQ-CS plays a crucial role in promoting the holistic physical and mental wellbeing of college students. Colleges may incorporate PL scores into students' physical and mental health assessments or utilize them for the National Student Physical Fitness Monitoring Program (Durden-Myers and Bartle, 2023). The cultivation of PL among college students should be integrated into the philosophy of higher education institutions. University education should emphasize the holistic development of students, encompassing multiple dimensions, such as competencies, affective attitudes, and moral character. Applying this questionnaire to teaching practices and instructional evaluations facilitates the integration of PL into educational objectives, enabling physical education to align with other academic disciplines and collectively promote students' holistic development. For example, in physical education classes, ideological and political content can be integrated with the learning of athletic skills to cultivate students' moral qualities such as adherence to rules and teamwork spirit. Students can use these metrics to identify their strengths and weaknesses in various areas, enabling them to develop personalized training plans and set tailored development goals. Meanwhile, the PLAQ-CS scores provide timely feedback on students' physical education learning progress. Physical education teachers can adjust curriculum content and teaching methods based on the weighting of each factor within the assessment criteria. For example, if a college student finds that they score highly in physical skills but lack collaborative communication abilities, they can focus on developing teamwork skills.

Enhancing college students' PL is one way of maintaining a healthy lifestyle (Blain et al., 2020). Understanding the specific circumstances of college students' PL not only helps enhance civic literacy and strengthen social responsibility but also promotes social harmony and stability while improving the quality of life of college students. Research indicates that PL may even influence life satisfaction in adulthood and later in life (Lloyd et al., 2024). Research also indicates the importance of PL in buffering the decline in college students' quality of life. Specifically, the interaction between physical competence, physical motivation, and physical fitness is closely associated with a higher quality of life. Furthermore, cultivating PL promotes lifelong participation in physical activities and enhances personal wellbeing, even during COVID-19 pandemic restrictions (Dong et al., 2023). The PLAQ-CS scores can enhance citizens' understanding of PL and actively cultivate their own PL proficiency. College students who actively participate in various sports activities can better integrate into society and enhance their communication skills and social adaptability, which will help reduce the occurrence of social conflict and tension. For example, college students can participate in community sports activities, foster neighborhood relationships through various athletic events, and create a positive social atmosphere.

### 4.4 Limitations

This study had two main limitations. First, the questionnaire developed here was primarily designed for college students, limiting its applicability to other populations (e.g., children and older adults). Only Chinese college students were interviewed, resulting in a relatively small and homogenous sample. To enhance the generalizability of the questionnaire, future studies could expand both the sample size and the demographic diversity. Second, the expertise involved in this study is limited to Chinese scholars. At the outset of the questionnaire development, only Chinese scholars were consulted, with no input from experts from other countries or regions. To strengthen the content reliability and validity of the questionnaire, future research should incorporate a more diverse pool of expert interviews. Meanwhile, to present the weighting scores for each domain of the PLAQ-CS more clearly, the Delphi method and Analytic Hierarchy Process (AHP) could be used in the future to enhance explanatory clarity.

## 5 Conclusions

The international development of PL assessment tools has emerged as a crucial strategy for promoting human health and enhancing overall wellbeing. The advancement of PL assessment instruments tailored for diverse populations—including children, adolescents, adults, and older adults—holds significant importance. This study utilized a mixed-methods research approach to create the PLAQ-CS. The PLAQ-CS comprises six primary assessment indicators, fifteen secondary assessment indicators, and forty-seven assessment items. It has demonstrated adequate reliability and validity, consistent with measurement science standards, thus establishing it as an appropriate tool for evaluating PL among college students. However, while the PLAQ-CS serves as a valuable resource for assessing and improving PL in this demographic, its universal applicability may be limited by variations in students' physical and psychological characteristics, as well as differences in educational systems. Therefore, future applications of the PLAQ-CS across various countries and populations will require revalidation, particularly within the context of physical education in schools.

## Data Availability

The original contributions presented in the study are included in the article/Supplementary material, further inquiries can be directed to the corresponding author.
