# Supplementary material for: Development and validation of a physical literacy assessment questionnaire for college students
Source: Front Psychol. 2026 Jan 30;16:1676038. doi: 10.3389/fpsyg.2025.1676038 (PMC12903775; doi:10.3389/fpsyg.2025.1676038)
Supplement: Supplementary file 1 [file Supplementary_file_1.docx]

**Appendix**

APPENDIX 1 Initial Physical Literacy Assessment Questionnaire for College Students

| Domains | Second  dimensions | Items | Degree of agreement |
| --- | --- | --- | --- |
| (A)PS | A1 FSK | A11 I can naturally participate in sports technique drills or games during physical education classes. | 1 2 3 4 5 |
|  |  | A12 I can perform basic walking, running, jumping, throwing and bracing movements. | 1 2 3 4 5 |
|  |  | A13 I can identify sports terminology describing changes in direction, speed variations, and force levels. | 1 2 3 4 5 |
|  |  | A14 My physical activity capacity is strong. | 1 2 3 4 5 |
|  |  | A15 I demonstrated strong athletic performance during physical activities. | 1 2 3 4 5 |
|  | A2 SSK | A21 I have mastered an athletic skill (not limited to multi-sport items such as soccer, basketball, etc.). | 1 2 3 4 5 |
|  |  | A22 I know the practice methods for a specific sports technique. | 1 2 3 4 5 |
|  |  | A23 I can apply mastered sports techniques scientifically and appropriately during competitions. | 1 2 3 4 5 |
|  |  | A24 I can explain the key points of certain sports techniques to peers. | 1 2 3 4 5 |
|  |  | A25 I can evaluate whether certain sports techniques are performed correctly. | 1 2 3 4 5 |
|  |  | A26 I understand the competition rules for certain sports. | 1 2 3 4 5 |
|  |  | A27 I can score good or above on sports techniques I have learned in physical education exams. | 1 2 3 4 5 |
| (B)PC | B1 PKAC | B11 I can learn sports knowledge through channels like fitness apps and short videos. | 1 2 3 4 5 |
|  |  | B12 I actively seek out and study sports knowledge. | 1 2 3 4 5 |
|  |  | B13 In physical education classes, I always pay close attention to the knowledge taught by the instructor. | 1 2 3 4 5 |
|  |  | B14 I understand the health risks of improper exercise techniques. | 1 2 3 4 5 |
|  |  | B15 I proactively learn the rules of sports events. | 1 2 3 4 5 |
|  | B2 PKC | B21 I clearly understand the requirements of physical fitness testing standards. | 1 2 3 4 5 |
|  |  | B22 I can articulate the benefits of participating in sports. | 1 2 3 4 5 |
|  |  | B23 I understand referees' decisions during sports competitions. | 1 2 3 4 5 |
|  |  | B24 I know appropriate exercise intensity should be maintained during physical activity. | 1 2 3 4 5 |
|  |  | B25 I understand the need to avoid exercising in unhealthy environments (e.g., smoggy days). | 1 2 3 4 5 |
|  |  | B26 I can reflect on technical or tactical mistakes made by myself or my team during competitions. | 1 2 3 4 5 |
|  | B3 PKAP | B31 I can apply learned sports knowledge to daily exercise routines. | 1 2 3 4 5 |
|  |  | B32 I can proactively organize or participate in sports competitions both on and off campus. | 1 2 3 4 5 |
|  |  | B33 I am able to apply 1-2 skills scientifically and rationally to improve cardiorespiratory endurance, muscular strength and flexibility (aerobic running, deep squats, etc.). | 1 2 3 4 5 |
|  |  | B34 I prioritize developing healthy lifestyle habits in daily life. | 1 2 3 4 5 |
|  |  | B35 I can create exercise plans for family members or classmates. | 1 2 3 4 5 |
|  |  | B36 I can properly address sports injuries. | 1 2 3 4 5 |
| (C)PA | C1 PMO | C11 I want to master a motor skill through my physical education class. | 1 2 3 4 5 |
|  |  | C12 A big reason I work hard in class is to get good grades. | 1 2 3 4 5 |
|  |  | C13 I work hard to improve my level of motor skills to be praised by my teachers and peers. | 1 2 3 4 5 |
|  |  | C14 When I participate in sports, I feel happy. | 1 2 3 4 5 |
|  |  | C15 I often engage in physical activities to cultivate my own interests and hobbies. | 1 2 3 4 5 |
|  |  | C16 I hope to make new friends by participating in sports activities. | 1 2 3 4 5 |
|  | C2 PCO | C21 I don't worry about quizzes in my physical education class. | 1 2 3 4 5 |
|  |  | C22 In physical education competitions, I think I can perform well. | 1 2 3 4 5 |
|  |  | C23 I am not afraid to participate in a variety of activities related to physical education and sports. | 1 2 3 4 5 |
|  |  | C24 Through learning, I believe I can master the sports skills I study. | 1 2 3 4 5 |
|  |  | C25 I believe I will come to enjoy sports. | 1 2 3 4 5 |
| (D)PH | D1 HS | D11 If nothing else, sports is something I have to do. | 1 2 3 4 5 |
|  |  | D12 I play sport even when there is no physical education class (or exam). | 1 2 3 4 5 |
|  |  | D13 I find a way to play sport even when it is difficult.  D14 I engage in physical activity three or more times per week. | 1 2 3 4 5  1 2 3 4 5 |
|  |  | D15 I can stick with the sports I enjoy. | 1 2 3 4 5 |
|  |  | D16 I exercise regardless of whether it's cold or hot outside. | 1 2 3 4 5 |
|  | D2 HA | D21 Sports are a part of my daily life. | 1 2 3 4 5 |
|  |  | D22 I don't need anyone's (teachers, parents) urging to participate in sport. | 1 2 3 4 5 |
|  |  | D23 In daily life, I consider physical activity perfectly normal. | 1 2 3 4 5 |
|  |  | D24 If I skip sport, my body feels uncomfortable. | 1 2 3 4 5 |
|  |  | D25 Whenever I have free time, I engage in physical activity. | 1 2 3 4 5 |
| (E)PM | E1 PSP | E11 I am not afraid of the sport challenges in classroom learning. | 1 2 3 4 5 |
|  |  | E12 I always persevere when I encounter difficulties or bottlenecks in learning sports skills. | 1 2 3 4 5 |
|  |  | E13 I always do my best when participating in sports. | 1 2 3 4 5 |
|  |  | E14 In athletic competitions, I will fight hard to achieve victory. | 1 2 3 4 5 |
|  |  | E15 I will strive to overcome difficulties encountered during sports activities. | 1 2 3 4 5 |
|  | E2 PE | E21 I follow the rules when participating in all kinds of sports. | 1 2 3 4 5 |
|  |  | E22 I can be honest in taking tests and do not cheat during sports tests. | 1 2 3 4 5 |
|  |  | E23 I think it is not right to cheat in sports competitions. | 1 2 3 4 5 |
|  |  | E24 When playing sports, I can show respect for my peers and opponents. | 1 2 3 4 5 |
|  |  | E25 Each time I exercise, I strictly adhere to and complete the planned workout. | 1 2 3 4 5 |
|  | E3 PCH | E31 I will take the initiative to help my classmates with poorer athletic performance to learn. | 1 2 3 4 5 |
|  |  | E32 Whether I win or lose, I look at the results of the game correctly. | 1 2 3 4 5 |
|  |  | E33 In sports activities, I get along well with classmates. | 1 2 3 4 5 |
|  |  | E34 In physical education classes, I strive to honor my team. | 1 2 3 4 5 |
|  |  | E35 When my mistakes disadvantaged the team, I actively took responsibility and implemented corrective measures. | 1 2 3 4 5 |
| (F)PF | F1 PQ | F11 I have never felt powerless when playing sports. | 1 2 3 4 5 |
|  |  | F12 I can react quickly when facing unexpected problems during sports. | 1 2 3 4 5 |
|  |  | F13 I find that my body has become more coordinated after I learn in the sports classroom. | 1 2 3 4 5 |
|  |  | F14 My exercise sessions consistently lasted for 1 hour or longer. | 1 2 3 4 5 |
|  |  | F15 In fitness assessments, my forward bend test consistently meets the “good” standard or higher. | 1 2 3 4 5 |
|  | F2 PFU | F21 I have never been unresponsive after I have played sports. | 1 2 3 4 5 |
|  |  | F22 I have never experienced fainting while playing sports. | 1 2 3 4 5 |
|  |  | F23 After participating in sports, I find that my immunity has improved (e.g., I get sick less often). | 1 2 3 4 5 |
|  |  | F24 My systolic and diastolic blood pressure fall within the normal range. | 1 2 3 4 5 |
|  |  | F25 After each workout, my body recovers quickly to its pre-exercise state (e.g., heart rate). | 1 2 3 4 5 |
|  |  | F26 My lung capacity meets or exceeds the “good” standard in physical fitness tests. | 1 2 3 4 5 |
|  | F3 PFO | F31 My body mass index (BMI) is within the standard range (BMI = weight (kg)/height (m)^2^ [18.5 ≤ standard ≤ 23.9]). | 1 2 3 4 5 |
|  |  | F32 I am satisfied with my physical form. | 1 2 3 4 5 |
|  |  | F33 I consider my physical form to meet the requirements of the sports I practice. | 1 2 3 4 5 |
|  |  | F34 During sport, I consider my physical form capable of supporting me in completing technical movements. | 1 2 3 4 5 |
|  |  | F35 I feel my physical shape imposes a burden during exercise. | 1 2 3 4 5 |

*A = Physical skill, B = Physical cognition, C = Physical affectivity, D = Physical habit, E = Physical morality, F = Physical fitness. A1 = Fundamental sport skills, A2 = Specialized sport skills, B1 = Physical knowledge acquisition, B2 = Physical knowledge comprehension, B3 = Physical knowledge application, C1 = Physical motivation, C2 = Physical confidence, D1 = Habit stabilization, D2 = Habit automation, E1 = Physical spirit, E2 = Physical ethics, E3 = Physical character, F1 = Physical quality, F2 = Physical functioning, F3 = Physical form.

APPENDIX 2 Expert Questionnaire

| Domains | Second  dimensions | Items | Degree of agreement |
| --- | --- | --- | --- |
| (A)PS | A1 FSK | A11 I can naturally participate in sports technique drills or games during physical education classes. | Agree / Disagree |
|  |  | A12 I can perform basic walking, running, jumping, throwing and bracing movements. | Agree / Disagree |
|  |  | A13 I can identify sports terminology describing changes in direction, speed variations, and force levels. | Agree / Disagree |
|  |  | A14 My physical activity capacity is strong. | Agree / Disagree |
|  |  | A15 I demonstrated strong athletic performance during physical activities. | Agree / Disagree |
|  | A2 SSK | A21 I have mastered an athletic skill (not limited to multi-sport items such as soccer, basketball, etc.). | Agree / Disagree |
|  |  | A22 I know the practice methods for a specific sports technique. | Agree / Disagree |
|  |  | A23 I can apply mastered sports techniques scientifically and appropriately during competitions. | Agree / Disagree |
|  |  | A24 I can explain the key points of certain sports techniques to peers. | Agree / Disagree |
|  |  | A25 I can evaluate whether certain sports techniques are performed correctly. | Agree / Disagree |
|  |  | A26 I understand the competition rules for certain sports. | Agree / Disagree |
|  |  | A27 I can score good or above on sports techniques I have learned in physical education exams. | Agree / Disagree |
| (B)PC | B1 PKAC | B11 I can learn sports knowledge through channels like fitness apps and short videos. | Agree / Disagree |
|  |  | B12 I actively seek out and study sports knowledge. | Agree / Disagree |
|  |  | B13 In physical education classes, I always pay close attention to the knowledge taught by the instructor. | Agree / Disagree |
|  |  | B14 I understand the health risks of improper exercise techniques. | Agree / Disagree |
|  |  | B15 I proactively learn the rules of sports events. | Agree / Disagree |
|  | B2 PKC | B21 I clearly understand the requirements of physical fitness testing standards. | Agree / Disagree |
|  |  | B22 I can articulate the benefits of participating in sports. | Agree / Disagree |
|  |  | B23 I understand referees' decisions during sports competitions. | Agree / Disagree |
|  |  | B24 I know appropriate exercise intensity should be maintained during physical activity. | Agree / Disagree |
|  |  | B25 I understand the need to avoid exercising in unhealthy environments (e.g., smoggy days). | Agree / Disagree |
|  |  | B26 I can reflect on technical or tactical mistakes made by myself or my team during competitions. | Agree / Disagree |
|  | B3 PKAP | B31 I can apply learned sports knowledge to daily exercise routines. | Agree / Disagree |
|  |  | B32 I can proactively organize or participate in sports competitions both on and off campus. | Agree / Disagree |
|  |  | B33 I am able to apply 1-2 skills scientifically and rationally to improve cardiorespiratory endurance, muscular strength and flexibility (aerobic running, deep squats, etc.). | Agree / Disagree |
|  |  | B34 I prioritize developing healthy lifestyle habits in daily life. | Agree / Disagree |
|  |  | B35 I can create exercise plans for family members or classmates. | Agree / Disagree |
|  |  | B36 I can properly address sports injuries. | Agree / Disagree |
| (C)PA | C1 PMO | C11 I want to master a motor skill through my physical education class. | Agree / Disagree |
|  |  | C12 A big reason I work hard in class is to get good grades. | Agree / Disagree |
|  |  | C13 I work hard to improve my level of motor skills to be praised by my teachers and peers. | Agree / Disagree |
|  |  | C14 When I participate in sports, I feel happy. | Agree / Disagree |
|  |  | C15 I often engage in physical activities to cultivate my own interests and hobbies. | Agree / Disagree |
|  |  | C16 I hope to make new friends by participating in sports activities. | Agree / Disagree |
|  | C2 PCO | C21 I don't worry about quizzes in my physical education class. | Agree / Disagree |
|  |  | C22 In physical education competitions, I think I can perform well. | Agree / Disagree |
|  |  | C23 I am not afraid to participate in a variety of activities related to physical education and sports. | Agree / Disagree |
|  |  | C24 Through learning, I believe I can master the sports skills I study. | Agree / Disagree |
|  |  | C25 I believe I will come to enjoy sports. | Agree / Disagree |
| (D)PH | D1 HS | D11 If nothing else, sports is something I have to do. | Agree / Disagree |
|  |  | D12 I play sport even when there is no physical education class (or exam). | Agree / Disagree |
|  |  | D13 I find a way to play sport even when it is difficult.  D14 I engage in physical activity three or more times per week. | Agree / Disagree  Agree / Disagree |
|  |  | D15 I can stick with the sports I enjoy. | Agree / Disagree |
|  |  | D16 I exercise regardless of whether it's cold or hot outside. | Agree / Disagree |
|  | D2 HA | D21 Sports are a part of my daily life. | Agree / Disagree |
|  |  | D22 I don't need anyone's (teachers, parents) urging to participate in sport. | Agree / Disagree |
|  |  | D23 In daily life, I consider physical activity perfectly normal. | Agree / Disagree |
|  |  | D24 If I skip sport, my body feels uncomfortable. | Agree / Disagree |
|  |  | D25 Whenever I have free time, I engage in physical activity. | Agree / Disagree |
| (E)PM | E1 PSP | E11 I am not afraid of the sport challenges in classroom learning. | Agree / Disagree |
|  |  | E12 I always persevere when I encounter difficulties or bottlenecks in learning sports skills. | Agree / Disagree |
|  |  | E13 I always do my best when participating in sports. | Agree / Disagree |
|  |  | E14 In athletic competitions, I will fight hard to achieve victory. | Agree / Disagree |
|  |  | E15 I will strive to overcome difficulties encountered during sports activities. | Agree / Disagree |
|  | E2 PE | E21 I follow the rules when participating in all kinds of sports. | Agree / Disagree |
|  |  | E22 I can be honest in taking tests and do not cheat during sports tests. | Agree / Disagree |
|  |  | E23 I think it is not right to cheat in sports competitions. | Agree / Disagree |
|  |  | E24 When playing sports, I can show respect for my peers and opponents. | Agree / Disagree |
|  |  | E25 Each time I exercise, I strictly adhere to and complete the planned workout. | Agree / Disagree |
|  | E3 PCH | E31 I will take the initiative to help my classmates with poorer athletic performance to learn. | Agree / Disagree |
|  |  | E32 Whether I win or lose, I look at the results of the game correctly. | Agree / Disagree |
|  |  | E33 In sports activities, I get along well with classmates. | Agree / Disagree |
|  |  | E34 In physical education classes, I strive to honor my team. | Agree / Disagree |
|  |  | E35 When my mistakes disadvantaged the team, I actively took responsibility and implemented corrective measures. | Agree / Disagree |
| (F)PF | F1 PQ | F11 I have never felt powerless when playing sports. | Agree / Disagree |
|  |  | F12 I can react quickly when facing unexpected problems during sports. | Agree / Disagree |
|  |  | F13 I find that my body has become more coordinated after I learn in the sports classroom. | Agree / Disagree |
|  |  | F14 My exercise sessions consistently lasted for 1 hour or longer. | Agree / Disagree |
|  |  | F15 In fitness assessments, my forward bend test consistently meets the “good” standard or higher. | Agree / Disagree |
|  | F2 PFU | F21 I have never been unresponsive after I have played sports. | Agree / Disagree |
|  |  | F22 I have never experienced fainting while playing sports. | Agree / Disagree |
|  |  | F23 After participating in sports, I find that my immunity has improved (e.g., I get sick less often). | Agree / Disagree |
|  |  | F24 My systolic and diastolic blood pressure fall within the normal range. | Agree / Disagree |
|  |  | F25 After each workout, my body recovers quickly to its pre-exercise state (e.g., heart rate). | Agree / Disagree |
|  |  | F26 My lung capacity meets or exceeds the “good” standard in physical fitness tests. | Agree / Disagree |
|  | F3 PFO | F31 My body mass index (BMI) is within the standard range (BMI = weight (kg)/height (m)^2^ [18.5 ≤ standard ≤ 23.9]). | Agree / Disagree |
|  |  | F32 I am satisfied with my physical form. | Agree / Disagree |
|  |  | F33 I consider my physical form to meet the requirements of the sports I practice. | Agree / Disagree |
|  |  | F34 During sport, I consider my physical form capable of supporting me in completing technical movements. | Agree / Disagree |
|  |  | F35 I feel my physical shape imposes a burden during exercise. | Agree / Disagree |
| *A = Physical skill, B = Physical cognition, C = Physical affectivity, D = Physical habit, E = Physical morality, F = Physical fitness. A1 = Fundamental sport skills, A2 = Specialized sport skills, B1 = Physical knowledge acquisition, B2 = Physical knowledge comprehension, B3 = Physical knowledge application, C1 = Physical motivation, C2 = Physical confidence, D1 = Habit stabilization, D2 = Habit automation, E1 = Physical spirit, E2 = Physical ethics, E3 = Physical character, F1 = Physical quality, F2 = Physical functioning, F3 = Physical form. | | | |
| Your suggestions: | | | |

APPENDIX 3 Expert Interview Suggestions

| Items | Expert suggestions | Percentage of expert in  agreement |
| --- | --- | --- |
| A14) My physical activity capacity is strong. | 1.Some items are not clearly defined, such as “My physical activity capacity is strong”. How is “strong” defined? | 64.3%  71.4% |
|  | 2. It is recommended to revise this ambiguous wording.  3. The physical activity abilities of different students vary, and the standard of “strong” is difficult to define.  4. Individuals have different criteria for assessing their own physical activity capabilities, and this should not be included as part of the test content. |  |
|  | 5. This question item may mislead respondents, preventing them from accurately assessing their actual circumstances. |  |
| A22) I know the practice methods for a specific sports technique. | 1.Some items, such as “I know the practice methods for a specific sports technique,” are more akin to content in the field of physical cognition.  2. This option should be placed under the “physical cognition” domain.  3. Does this question item assess knowledge or applied skills? Do not overlap.  4. This item does not belong to the skill domain. |  |
| A23) I can apply mastered sports techniques scientifically and appropriately during competitions. | 1. Whether the application of sports techniques is reasonable or not, the requirements for each person are different.  2. Suggest deleting vague expressions.  3. Students may not be able to understand such items.  4. The scientific and rational nature of sports techniques cannot be measured by language. | 71.4% |
| B14) I understand the health risks of improper exercise techniques. | 1. This item should not be placed within the dimension of physical knowledge acquisition.  2. It is important to distinguish between “knowledge acquisition” and “knowledge comprehension.”  3. This item requires stronger wording, as it currently overlaps with other dimensions.  4. It is recommended to adjust the position of this item. | 71.4% |
| B26) I can reflect on technical or tactical mistakes made by myself or my team during competitions. | 1. The content of items in B2, such as B26, shows some overlap with the content in B3 “physical knowledge application.”  2. It is important to distinguish between the two, as this phrasing can easily lead to misunderstanding.  3. It is recommended that the content of this item be revised.  4. Reflection should occur at a higher level than understanding. | 71.4% |
| D14) I engage in physical activity three or more times per week. | 1. Do not include content that may raise suspicion. What is the scientific basis for claiming that exercising three times a week represents a stable habit?  2. This standard does not apply to everyone.  3. This item requires revision, as exercising three times a week does not demonstrate the stability of the habit.  4. It is recommended that such quantitative content be revised unless there is clear justification.  5. Delete this question item.  6. Due to individual variations in physical constitution, the number “3” may not accurately reflect the meaning of dimension D1. | 57.1% |
| F14) My exercise sessions consistently lasted for 1 hour or longer. | 1. Remove the description of the scale content.  2. This standard does not apply to all students.  3. This has the same problem as D14.  4. Refine the content of this question item.  5. What is the scientific basis for this item? | 64.3% |
| F15) In fitness assessments, my forward bend test consistently meets the “good” standard or higher. | 1. This item is not recommended for use. Since the physical fitness test content is part of the Chinese physical education curriculum assessment, it would undermine the value of the scale.  2. If such an item exists, simply use the indicators from the physical fitness test.  3. Flexibility does not necessarily have to be assessed using the forward bend test.  4. Why not use the indicators from the physical fitness test? | 71.4% |
| F24) My systolic and diastolic blood pressure fall within the normal range. | 1. Physiological indicators generally require on-site testing, relying solely on students' recollections can lead to significant inaccuracies.  2. Many students may not accurately know their blood pressure readings.  3. This item can easily confuse students because they may not be clear on the normal range for blood pressure.  4. If on-site measurement is not feasible, it is recommended to remove this item. | 71.4% |
| F26) My lung capacity meets or exceeds the “good” standard in physical fitness tests. | 1. This item shares the same issue as D14 and F14.  2. The lung capacity metric is not recommended for inclusion in the scale, as it is inherently a quantitative indicator.  3. For the validity of the scale, this item needs to be deleted.  4. Physical fitness test indicators can be used directly without the need for measurement scales. | 71.4% |
